# Wireless Sensor Networks for Environmental Monitoring

**DOI:** 10.3390/s21041172

**Published:** 2021-02-07

**Authors:** Anna Lanzolla, Maurizio Spadavecchia

**Affiliations:** Department of Electrical and Information Engineering, Politecnico di Bari, 70126 Bari, Italy; maurizio.spadavecchia@poliba.it

In this editorial, an overview of the content of the Special Issue on “Wireless Sensor Networks for Environmental Monitoring” is provided. The aim of this Special Issue is to survey and discuss the state of the art, difficulties, innovations, and improvements on environmental data acquisition, monitoring, analysis, and risk assessment and management.

Wireless sensor networks (WSNs) allow for innovative and attractive solutions, as well as for pervasive environmental monitoring by providing many important benefits such as real time access to data, coverage of wide areas, long-term monitoring, and system scalability. These networks consist of a large number distributed devices, each including sensing, processing, and wireless communications capabilities, and their uses have greatly improved remote environmental sensing, the monitoring of several physical systems, and risk assessment and management.

A total of 11 papers were submitted to this Special Issue. After a careful peer review process, six of these were finally accepted for publication. In the following, a brief overview of the published papers is provided by pointing out the contribution of each paper in proposing innovative solutions in the field environmental monitoring based on the use of WSN.

Environmental monitoring is fundamental to understand our ecosystem in order to prevent adverse effects on human health and environment. Urban noise affects more than quality of life and can cause long-term physiological damage. In this context, wireless acoustic sensor networks (WANs) have become a promising solution to monitor urban noise and recognize acoustic events with a high performance. The study proposed in [1] provides a significant example of development of novel system that utilizes WASNs for the recording, transferring, post-processing, and recognition of urban noise in order to create and visualize noise maps of acoustic events, as well as to present additional information and noise statistics. Experimental results demonstrated that the proposed system can measure the urban noise and recognize acoustic events with a high performance in real-life scenarios.

The constant checking of plant health and the prompt detection of their pathogens are very important issues in environmental monitoring. Recent advances in technology, instrumentation, and procedures have created new opportunities for the real time monitoring and rapid and accurate identification of environmental disease. The paper [2] presents a remote sensing technique to monitor the spread of quick decline syndrome in olive trees caused by *Xylella fastidiosa* bacteria by using a multispectral camera mounted on a multirotor Unmanned Aerial Vehicle (UAV). The proposed method includes preprocessing to obtain calibrated reflectance images, the 3D reconstruction of a sparse cloud of points with stereophotogrammetry, the segmentation of trees, and the classification of their health status. The classifier was separately trained on each multispectral stack, but training on a whole set of homogenous stacks would further increase its reliability. This technique has the advantage of being potentially self-adapting to different kinds of trees and soil.

In addition to the monitoring of plant health status, examples of environmental studies on the identification of changes in coastal dunes induced by human activities are included in this Special Issue. In [3], the development of a low-cost and wireless wind data acquisition system that allowed for multiple synchronized wind measurement points enabled the data-intensive modelling of the interaction of an arid foredune system with its shrub vegetation. Given the scenario of global change, the monitoring of environmental aspects is very important in coastal areas, as many of them present aeolian sedimentary environments in arid zones that need special protection.

Deploying IoT sensors in WSNs is a crucial issue in network design and affects most important performance metrics like increasing coverage, strengthening connectivity, improving robustness, and increasing the lifetime Therefore, a sensor deployment method must be carefully designed to achieve such objective functions without exceeding the available budget. The authors of [4] introduced a novel deployment algorithm, based on Delaunay triangulation to find optimal locations for improving coverage while maintaining connectivity and robustness despite the existence of obstacles in the sensing area. The performance of the proposed method was evaluated in terms of area coverage, end-to-end-delay, and resilience to attacks, and it proved promising results. In addition, a dynamic network deployment method based on the hybrid hierarchical network was proposed in [5] to realize a low-cost, energy-saving, and real-time dynamic sensing system for overhead high-voltage transmission lines. The proposed method enhances the practicability of the network and improves the monitoring efficiency of the smart grid.

Another hot topic of WSNs is the connectivity among different nodes and systems; with the advent of the 5G technology, various types of massive wireless (IoT) services are becoming pervasive. In this scenario, the spectrum will be more and more congested, and frequency resources will thus become scarce. A possible solution could be the spectrum sharing supported by an automated frequency coordination system. In this direction in [6], the authors proposed a frequency coordination system capable of identifying whether a requested frequency band is eligible for spectrum sharing. A modified version of Kriging interpolation for the recapitulation of a radio environment map on the basis of the Support Vector Machine (SVM)-based variogram model was proposed. The proposed nonparametric modeling approach has a crucial role in making a confident decision regarding spectrum sharing and, therefore, in the future development of wider WSNs devoted to environmental monitoring.

The six papers reported in this Special Issue provide excellent examples of the current progress in the area of wireless sensor network applications for environmental monitoring. 

We think that the readers will consider this Special Section interesting, and its contents can contribute to new developments and results in scientific research. 

Thanks are due to all the authors for the time and effort they dedicated in preparing their highly interesting works, as well as to all the highly qualified reviewers for their valuable contributions to this Special Issue. 

We would also like to thank the editorial team at MDPI for its precious support and great help.
